# Radiologic Assessment of Malpositioned Indwelling Catheters and Medical Devices: A Retrospective Case Series With Pictorial Review

**DOI:** 10.7759/cureus.96127

**Published:** 2025-11-05

**Authors:** Arya Kermanshah, Daniela C Galan, Etienne C Gozlan, Ashmeet Bedi, Raghav Pai, Armaan M Sobhan, Carolina Padilla, Patricia Castillo

**Affiliations:** 1 Medical Education, University of Miami, Miami, USA; 2 Internal Medicine, St. John's Riverside Hospital, Yonkers, USA; 3 ER/Trauma and Acute Care Radiology, University of Miami Miller School of Medicine, Jackson Memorial Hospital, Miami, USA; 4 Urology, University of Miami Miller School of Medicine, Jackson Memorial Hospital, Miami, USA; 5 Medicine, Lake Erie College of Osteopathic Medicine, Erie, USA; 6 Radiology, University of Miami, Miami, USA; 7 Medicine, St. George's University School of Medicine, Boca Raton, USA; 8 Radiology, University of Miami Miller School of Medicine, Jackson Memorial Hospital, Miami, USA; 9 Radiology, University of Miami Miller School of Medicine, Miami, USA

**Keywords:** central venous catheter, device complications, ivc filter, medical device migration, misplaced catheter, nasogastric tube (ngt), peg tube, radiological evaluation, ureteral stent, ventriculoperitoneal shunt

## Abstract

Background: Indwelling medical devices such as central venous catheters (CVCs), ureteral stents, ventriculoperitoneal (VP) shunts, inferior vena cava (IVC) filters, percutaneous endoscopic gastrostomy (PEG) tubes, and nasogastric (NG) tubes are integral to modern clinical care. Malposition or migration, however, can result in severe complications, including vascular injury, organ perforation, infection, and obstruction. Radiologists are often the first to detect these complications, but interpretation may be challenging due to variable device types, insertion techniques, and patient anatomy.

Methods: We conducted a retrospective case-based review of malpositioned indwelling devices encountered in clinical practice at our institution. Each case was analyzed individually with attention to device type, insertion pathway, radiographic landmarks, abnormal positioning patterns, and associated complications. Representative examples were selected to create a pictorial review highlighting imaging pearls and diagnostic pitfalls.

Results: The cases illustrate a wide spectrum of malposition patterns across commonly placed devices. Vascular access complications included arterial cannulation, aberrant venous placement, and migration into unexpected vascular channels. Ureteral stent malpositions demonstrated distal migration into the urethra and proximal perforation through the renal cortex. VP shunt cases showed distal extrusion into hollow viscera such as the rectum. IVC filter malpositions included tilt, strut perforation, and extension into adjacent organs. PEG tube malpositions resulted in intraperitoneal leakage and peritonitis, while NG tube misplacements involved tracheobronchial insertion and gastric perforation. These examples highlight both typical and rare presentations, underscoring the importance of systematic radiographic evaluation.

Conclusion: This retrospective case series and pictorial review underscore the critical role of radiologists in recognizing malpositioned indwelling devices. Accurate interpretation requires a structured imaging approach, correlation with clinical context, and familiarity with device-specific pitfalls. By presenting illustrative examples across multiple device types, this work provides a practical reference to enhance radiologic vigilance, improve diagnostic accuracy, and ultimately prevent adverse outcomes in patients with indwelling catheters and medical devices.

## Introduction

Indwelling medical devices are essential tools in modern healthcare, playing roles in medication delivery, fluid management, nutritional support, and monitoring. While these devices are crucial, complications such as malpositioning and migration are not uncommon and may go unnoticed without imaging. In pediatric ICUs, prospective cohort data reveal complication rates of about 21%, many of which go unnoticed without deliberate surveillance [1]. Likewise, systematic reviews in adult critical care identify a central venous access device failure rate of approximately 5%, with dislodgement and malposition frequently leading to device removal and increased patient morbidity [2]. Radiologists play a central role in ensuring the safe and effective use of these devices through proper interpretation and recognition of abnormal placements.

This article provides a practical, evidence-based framework for evaluating commonly encountered indwelling devices, including central venous catheters (CVCs), ureteral stents, ventriculoperitoneal (VP) shunts, inferior vena cava (IVC) filters, percutaneous endoscopic gastrostomy (PEG) tubes, and nasogastric (NG) tubes. For each device, we review ideal positioning, key radiographic identification landmarks, and common misplacement patterns. Real clinical cases are included to highlight malposition and potential complications and reinforce interpretation strategies that can help prevent diagnostic delays and patient harm.

## Materials and methods

Methods

 All figures are original clinical images from the included cases.

Study design

This work was conducted as a retrospective, illustrative case series and pictorial review of malpositioned indwelling catheters and medical devices encountered in clinical practice. The aim was to assemble representative examples of device malposition and migration across multiple device types, accompanied by imaging findings and clinical context, in order to provide a practical framework for radiologic interpretation.

Case identification and selection

Cases were handpicked by senior radiology faculty from our institution’s archives (2020-2025), with selection based on their clinical relevance, educational value, and imaging clarity. Subspecialty radiologists with expertise in interventional, abdominal, and neuroimaging contributed cases they considered to be illustrative or diagnostically challenging examples of device malposition.

To ensure broad coverage, we sought to include at least one representative case for each major category of indwelling device commonly encountered in radiology practice: CVCs (internal jugular, subclavian, femoral, PICC lines, and ports), ureteral stents, VP shunts, IVC filters, PEG tubes, and NG tubes.

Inclusion criteria

Cases were selected if (1) imaging clearly demonstrated malposition or migration, (2) images were of diagnostic quality suitable for publication, and (3) patient consent for educational use was available.

Exclusion criteria

Cases were excluded if imaging quality was nondiagnostic, if device position could not be definitively determined, or if patient consent was unavailable.

When multiple examples of a given device type were available, selection was prioritized based on clarity of imaging (e.g., multimodality confirmation, presence of annotated landmarks), clinical significance of the complication (e.g., organ perforation vs. minor migration), and educational value for highlighting diagnostic pitfalls.

Imaging review

For each included case, all relevant imaging studies (radiographs, CT, MRI, or fluoroscopic images) were independently reviewed by at least two board-certified radiologists, including subspecialists as appropriate. Discrepancies in interpretation were resolved by consensus.

The following elements were systematically assessed: Device pathway and insertion approach (e.g., jugular vs. femoral for CVCs), expected anatomic landmarks for correct positioning, type and pattern of malposition or migration, associated complications (vascular injury, visceral perforation, obstruction, infection, etc.), comparison with prior imaging when available, to assess interval migration. Clinical data, including patient age, indication for device placement, and relevant outcomes, were extracted from the electronic medical record (EMR) when necessary to provide clinical context.

Data presentation

Cases were compiled into a pictorial review format. Each device section includes: A brief description of the correct positioning criteria, common malposition patterns from the literature, the selected illustrative case with annotated imaging, and a discussion of potential complications and diagnostic considerations.

Ethical considerations

All cases were anonymized to remove patient identifiers prior to analysis and publication. Institutional requirements regarding the educational use of imaging were followed. Patient consent for publication was obtained for each case included. Formal Institutional Review Board (IRB) approval was not required, as this study qualifies as an educational retrospective case series with no interventional component.

## Results

A spectrum of malpositioned indwelling devices was identified and compiled into this pictorial case series. Each case demonstrates a distinct mechanism of malposition or migration, representative of complications radiologists may encounter in practice.

Central venous catheters

Examples included arterial cannulation, aberrant venous placement into collateral veins, and cephalad migration via the lumbar venous plexus.

CVC: Upper Extremity Approach

**Figure 1:**
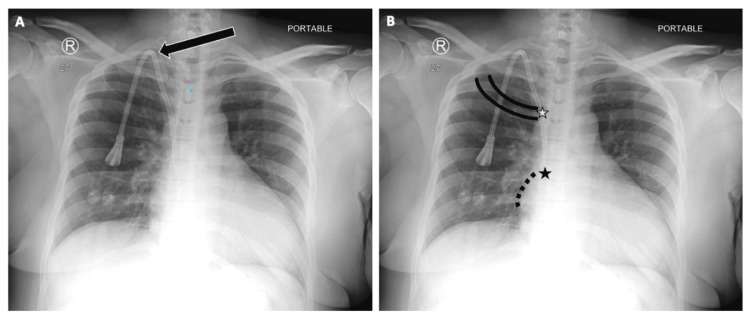
Chest Radiographs Demonstrating Proper Central Venous Catheter (CVC) Placement and Key Anatomical Landmarks Reference chest radiographs of upper extremity CVC placement. (A) Frontal chest radiograph showing a right internal jugular CVC (arrow) with the catheter tip correctly positioned at the cavoatrial junction. (B) Key anatomical landmarks used in CVC evaluation: intercostal spaces and rib margins for orientation; the superior vena cava (SVC, gray star); and the right atrial angle with cavoatrial junction (black star). Proper recognition of these landmarks is essential for confirming appropriate CVC tip location and avoiding malposition.

To assess positioning, begin by identifying the insertion site. A catheter coursing above the clavicle suggests internal jugular access, whereas one coursing below suggests subclavian access. On a chest radiograph, the SVC can be localized between the right first intercostal space and the superior margin of the right second rib (Figure 1). The cavoatrial junction, which is the ideal catheter tip location, is at the junction of the right atrium and mediastinum (Figure 1B).

Case Example

**Figure 2:**
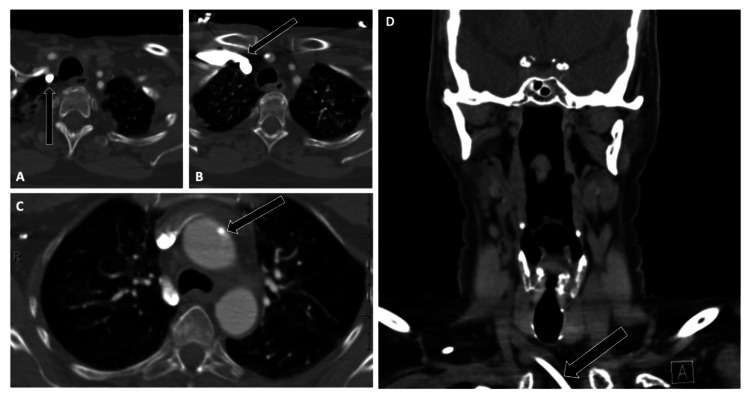
Arterial Malposition of a Central Venous Catheter on Chest CT Angiography Chest CT angiography demonstrating a chemotherapy catheter inadvertently placed within the arterial system. (A-C) Axial views show the catheter coursing through the right subclavian artery (A), continuing into the brachiocephalic artery (B), and terminating in the aortic arch (C) (arrows). (D) Coronal reconstruction confirms the arterial trajectory of the catheter (arrow). Recognition of this malposition is critical given the risk of catastrophic vascular complications.

A 72-year-old oncology patient underwent CT angiography for syncope during chemotherapy. Imaging revealed intra-arterial catheter placement extending from the subclavian artery to the aortic arch (Figure 2), an uncommon but high-risk malposition.

CVC: Lower Extremity Approach

**Figure 3:**
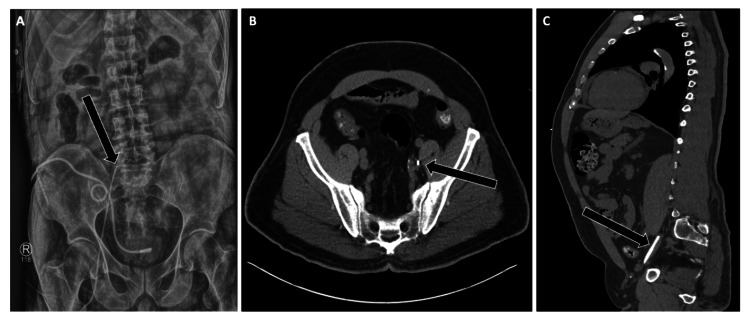
Correct Placement of a Femoral Central Venous Catheter Imaging examples of an appropriately positioned femoral central venous catheter. (A) KUB radiograph demonstrates the catheter tip projecting over the expected region of the iliac vein (arrow). (B-C) Axial and sagittal CT images (soft-tissue window) confirm catheter tip location within the common iliac vein/IVC (arrows). Recognition of these landmarks is essential to distinguish correct positioning from malposition into collateral or paraspinal venous pathways. IVC: Inferior vena cava

The tip of a femoral CVC should terminate in the common iliac vein or inferior vena cava (IVC) [3]. Radiographic confirmation begins by identifying the insertion site. If the catheter tip projects at the sacral alar, it is likely in the iliac vein; a projection near the right lumbar paravertebral region suggests IVC placement (Figure 3).

While malpositioning in adults is uncommon, it can occur, especially on the left side, due to anatomical angulation between the left iliac vein and ascending lumbar vein. In such cases, the catheter may enter the lumbar venous plexus and even traverse into the spinal canal.

Case Example

**Figure 4:**
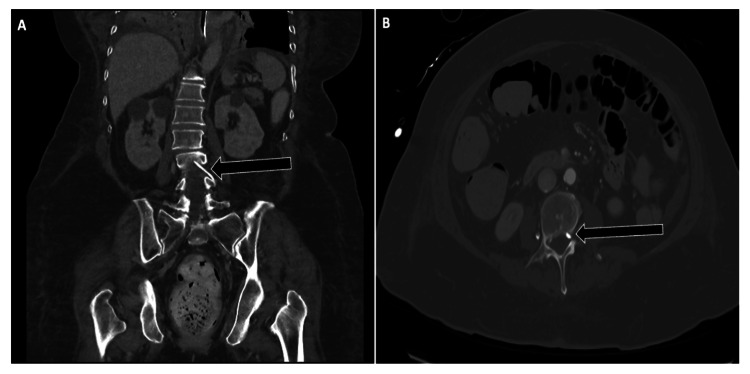
Unusual Femoral Catheter Malposition Into the Spinal Canal Non-contrast CT of the abdomen and pelvis demonstrating malposition of a left femoral venous catheter tracking into the spinal canal via collateral venous pathways. (A) Coronal reconstruction shows the catheter coursing cephalad through the lumbar venous system and traversing the L3-L4 neural foramen (arrow). (B) Axial image confirms entry into the anterior epidural venous plexus (arrow). This rare complication underscores the importance of careful review of catheter trajectory on cross-sectional imaging, particularly in left-sided femoral line placements.

An elderly trauma patient developed abdominal symptoms, prompting investigation. CT imaging revealed that a left femoral catheter had migrated cephalad via the lumbar vein, traversing the neural foramen into the spinal canal (Figure 4). This rare but serious misplacement underscores the need for heightened vigilance, particularly with left-sided femoral lines.

Ureteral stents

**Figure 5:**
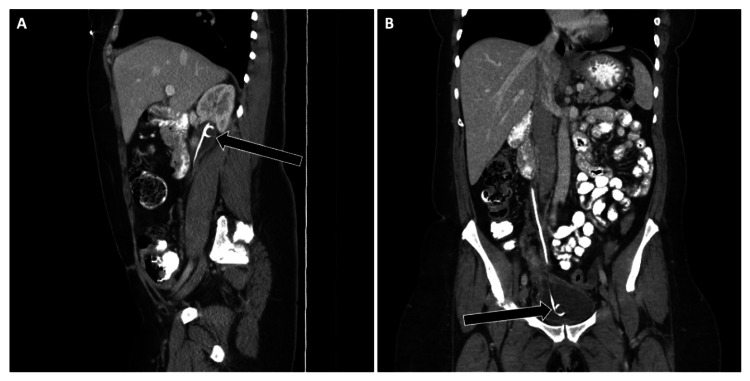
Correct Positioning of a Double-J Ureteral Stent Contrast-enhanced CT of the abdomen and pelvis demonstrating appropriate placement of a right double-J ureteral stent. (A) Sagittal reconstruction shows the proximal pigtail loop seated in the renal pelvis (arrow). (B) Coronal reconstruction demonstrates the distal pigtail loop appropriately coiled within the urinary bladder (arrow). Proper recognition of both proximal and distal positions is essential to confirm correct stent placement and exclude migration or perforation.

Cases showed both distal migration into the urethra and proximal perforation through the renal cortex with associated hematoma. Ideal positioning involves the proximal loop of the ureteral stent residing in the renal pelvis and the distal loop in the urinary bladder [4]. On KUB radiographs, the proximal loop should project medially to the renal silhouette, and the distal loop should appear within the lower pelvis (Figure 5).

Ureteral Stent Case Example 1

**Figure 6:**
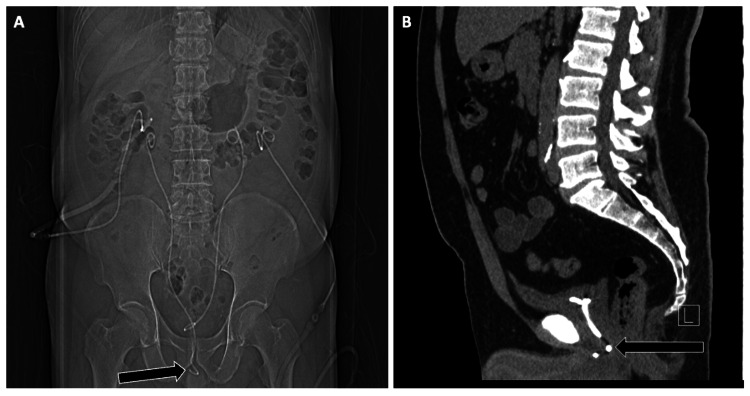
Distal Migration of a Ureteral Stent Into the Prostatic Urethra Imaging of a malpositioned right ureteral stent demonstrating distal migration. (A) Supine abdominal radiograph shows the stent tip projecting below the pubic symphysis (arrow). (B) Sagittal non-contrast CT confirms distal stent migration with the pigtail loop extending into the prostatic urethra (arrow). Recognition of the abnormal distal course is critical, as urethral migration can cause pain, hematuria, and urinary obstruction.

A patient with pelvic discomfort underwent KUB and CT, revealing distal migration of a right ureteral stent into the prostatic urethra (Figure 6).

Ureteral Stent Case Example 2

**Figure 7:**
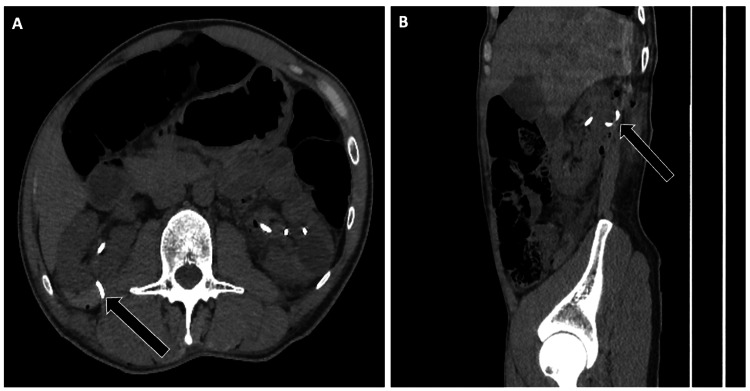
Proximal Malposition of a Ureteral Stent With Renal Cortex Perforation Non-contrast CT of the abdomen and pelvis demonstrating malposition of a right ureteral stent. (A) Axial image shows the proximal stent tip extending beyond the posterior renal cortex (arrow). (B) Sagittal reconstruction confirms extrarenal location of the stent tip with an associated perirenal hematoma (arrow). This complication highlights the importance of verifying stent position relative to the renal collecting system to avoid parenchymal injury.

A young male post-nephrolithotomy developed flank pain. CT showed cephalad migration of the proximal stent tip, which had perforated through the renal cortex, resulting in a perirenal hematoma (Figure 7).

Ventriculoperitoneal shunts

A typical shunt includes a proximal ventricular catheter, valve, reservoir, and distal catheter [5]. Malposition was illustrated by distal catheter extrusion into the rectum. Inferior vena cava filters: Complications included filter tilt and strut perforation extending into adjacent organs such as the duodenum. Percutaneous endoscopic gastrostomy tubes: Malposition resulted in intraperitoneal placement with peritoneal leakage and peritonitis.

**Figure 8:**
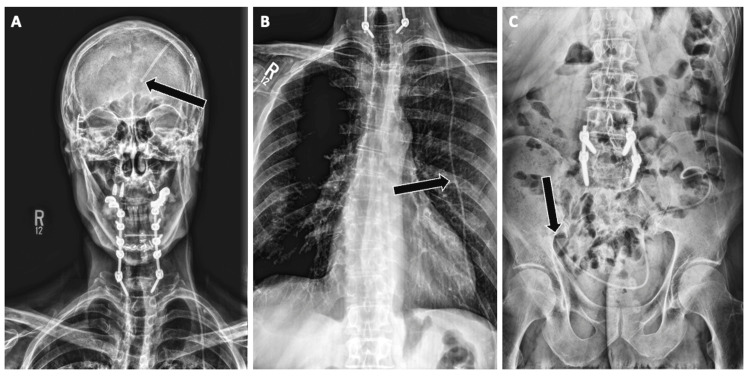
Radiographic Series Demonstrating Appropriate Ventriculoperitoneal (VP) Shunt Placement Radiographic evaluation of a properly positioned VP shunt. (A) Head and neck radiograph shows the proximal catheter tip within the lateral ventricle and its course descending through the neck (arrow). (B) Chest radiograph demonstrates the shunt traversing the mediastinum toward the abdomen (arrow). (C) Pelvic radiograph shows the distal catheter tip coiled appropriately within the peritoneal cavity (arrow). Sequential imaging across these regions is essential to confirm shunt integrity and appropriate positioning from ventricle to peritoneum.

The proximal tip of a VP shunt should reside in the frontal horn of a lateral ventricle, just anterior to the Foramen of Monro and away from the choroid plexus. The valve generally projects over the scalp, while the distal catheter courses subcutaneously through the neck, chest, and abdominal wall into the peritoneal cavity (Figure 8).

Imaging evaluation should verify the integrity and position of each component, from the lateral ventricles to the peritoneal cavity. The shunt tubing should appear continuous, without kinks, breaks, or disconnections. Radiographs are commonly used; however, CT scans may be required to assess for migration or potential obstruction.

Case Example

**Figure 9:**
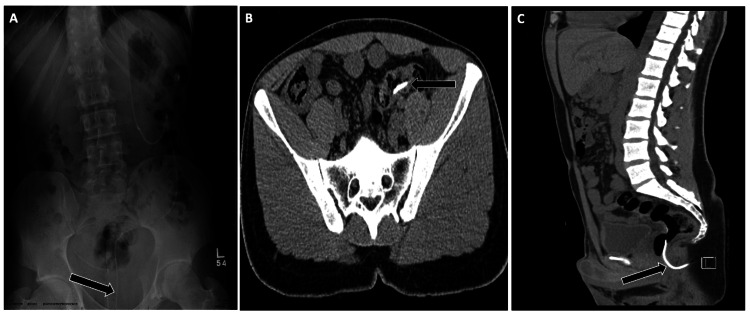
Distal Migration and Extrusion of a Ventriculoperitoneal (VP) Shunt Through the Anus Imaging of a Migrated VP Shunt with Distal Catheter Extrusion (A) Abdominal radiograph demonstrates the VP shunt catheter (arrow) projecting over the region of the descending/sigmoid colon and rectum, with the distal tip extending beyond the field of view. (B) Axial non-contrast CT image of the abdomen shows the catheter coursing through the sigmoid colon lumen (arrow). (C) Sagittal non-contrast CT of the abdomen and pelvis confirms the distal shunt tip traversing the rectum and extruding through the anus (arrow). This rare but serious complication carries a high risk of infection and requires urgent surgical management.

A young man with a childhood history of a brain tumor and VP shunt placement presented due to the presence of a catheter protruding from the anus. CT revealed distal shunt migration into the rectum with extrusion via the anus (Figure 9). Though rare, transanal shunt extrusion carries a high risk for infection and demands prompt surgical intervention.

Inferior vena cava filters

**Figure 10:**
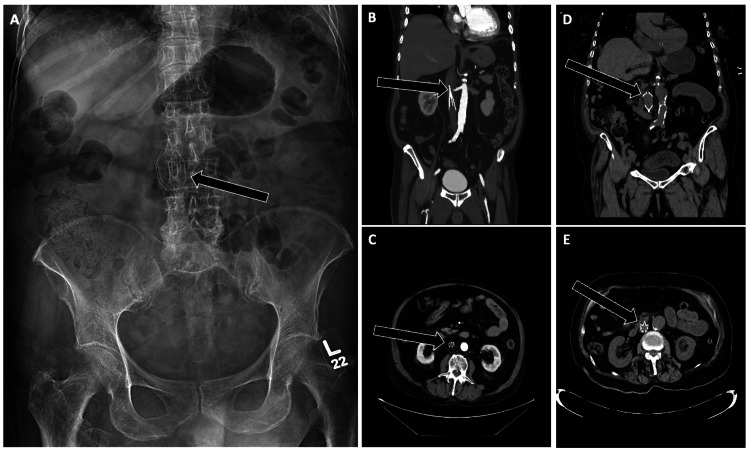
Imaging of Properly Positioned Inferior Vena Cava (IVC) Filters Imaging demonstrating appropriate positioning of various IVC filters. (A) Radiograph showing an “Optease” filter at the L3-L4 vertebral level (arrow). (B, C) Coronal and axial CT images of a “Greenfield” filter appropriately deployed within the infrarenal IVC (arrows). (D, E) Coronal and axial CT views of a “Mobin-Uddin” filter in the expected infrarenal position (arrows). Proper identification of filter type and verification of infrarenal positioning are essential to ensure effective protection against pulmonary embolism and to avoid complications such as tilt or caval penetration.

Correct placement is in the infrarenal IVC, verified through fluoroscopy or CT study. Venocavography, intravascular ultrasound, or duplex ultrasound may be used to evaluate the IVC anatomy prior to filter placement, ensuring the filter is properly located between the renal and iliac veins [6]. According to guidelines by the Society of Interventional Radiology and collaborating societies, the chosen imaging method should clearly delineate caval anatomy, measure the IVC diameter, identify the renal and iliac vein junctions, and detect any thrombus near the intended deployment site [7]. While fluoroscopic and intravascular ultrasound guidance are commonly used, current evidence does not support one technique over the other [7]. Final imaging must assess the orientation, position, and relationship of the IVC filters to nearby vascular landmarks (Figure 10).

Case Example

**Figure 11:**
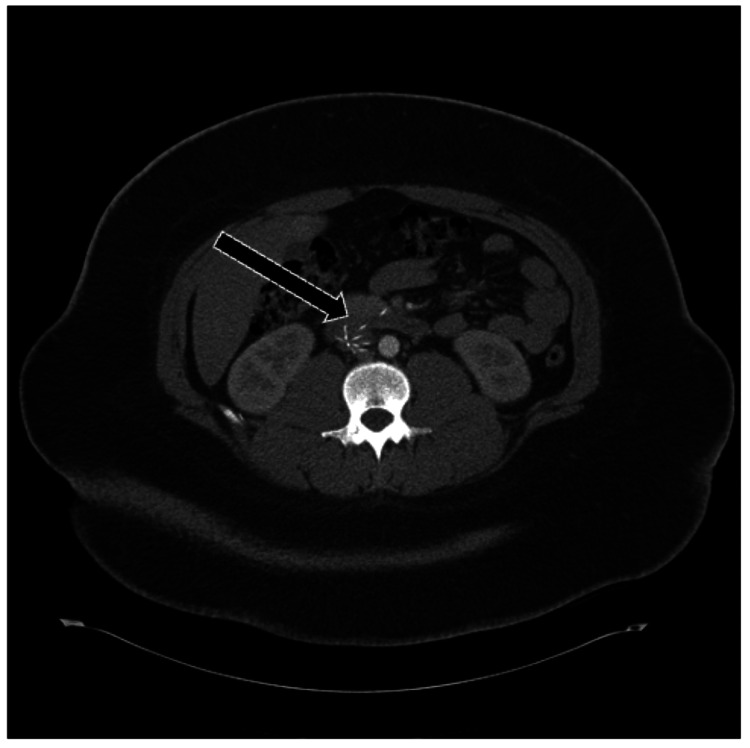
IVC Filter Complication: Strut Perforation Into the Duodenum Axial non-contrast CT of the abdomen demonstrating an infrarenal IVC filter complication. One of the filter struts is displaced, perforating the wall of the third portion of the duodenum (arrow). Recognition of adjacent organ perforation is critical, as it can lead to hemorrhage, infection, or perforation-related sequelae requiring urgent intervention.

A 48-year-old woman with abdominal pain underwent CT, revealing that a filter strut had perforated the third portion of the duodenum (Figure 11). This serious complication required prompt multidisciplinary evaluation. She underwent endovascular filter retrieval via a transjugular snare technique, followed by endoscopic evaluation of the duodenal wall. The patient was then managed conservatively with bowel rest and antibiotics.

Percutaneous endoscopic gastrostomy tubes

**Figure 12:**
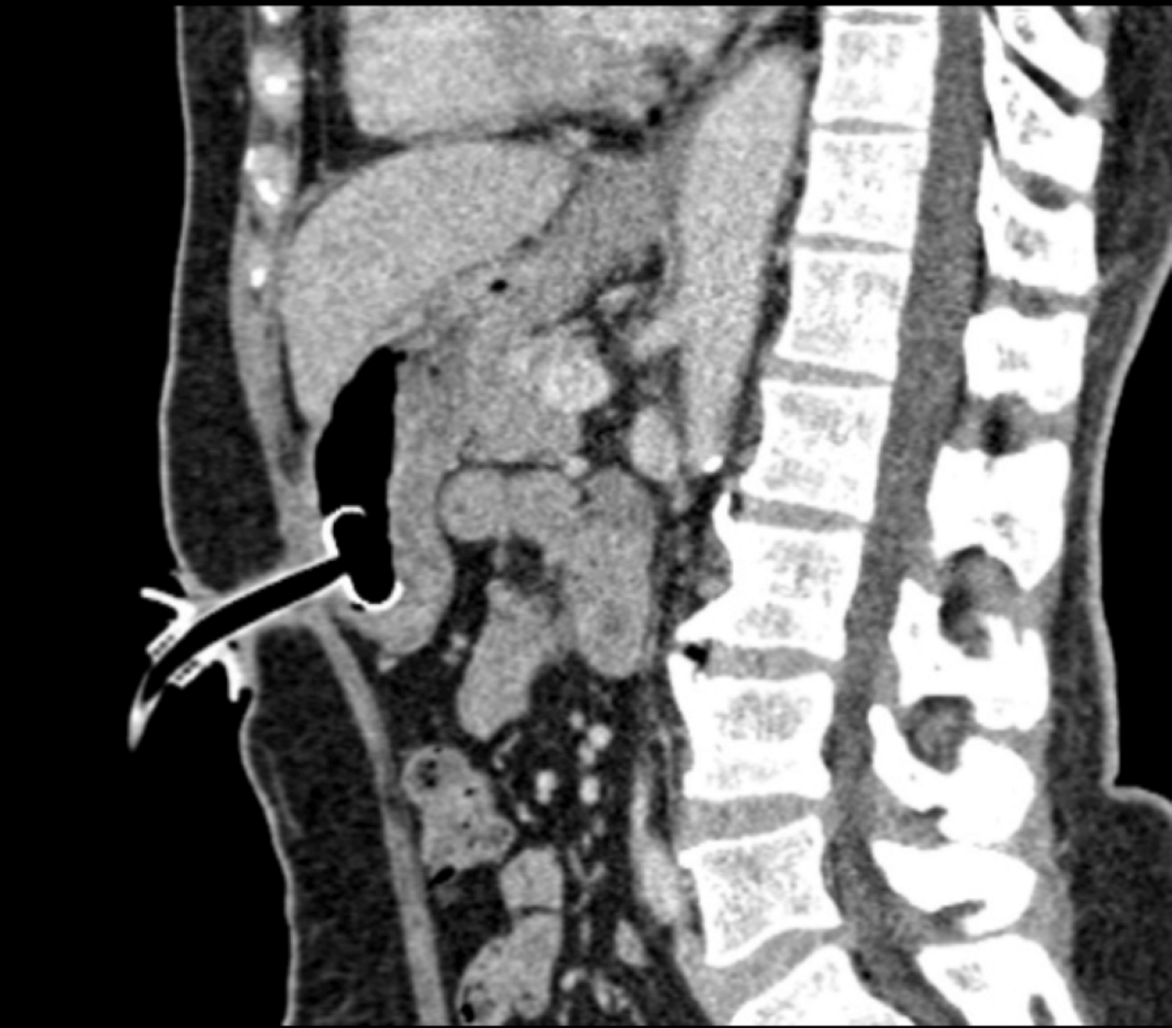
Correct Placement of a Percutaneous Gastrostomy Tube Sagittal non-contrast CT image demonstrating appropriate positioning of a percutaneous gastrostomy tube. The inflated retention balloon is located within the gastric body (arrow), confirming intragastric placement. Recognition of this appearance is essential to differentiate correct positioning from perigastric or intraperitoneal malposition.

Placement involves inserting a flexible tube through the anterior abdominal wall into the stomach, typically with a gastric balloon and external bumper to secure position [8]. Ideal placement positions the tube in the gastric body, between the lesser and greater curvatures, avoiding the left gastric and epiploic vessels. On KUB radiographs, the inflated balloon should project over the left upper or mid-abdomen (Figure 12). Placement is confirmed during the procedure via transillumination and finger indentation. A needle and guidewire are introduced, and the PEG tube is pulled through the GI tract and out through the abdominal wall for secure placement [9,10]. Follow-up imaging may include fluoroscopy, CT, or ultrasound, although fluoroscopy remains the gold standard for real-time positioning [11,12].

Minor complications include wound infections, peristomal leakage, and transient pneumoperitoneum. More serious complications include hemorrhage, perforation of adjacent organs, and rare cases of tumor seeding at the PEG site, particularly in head and neck cancers.

Case Example

**Figure 13:**
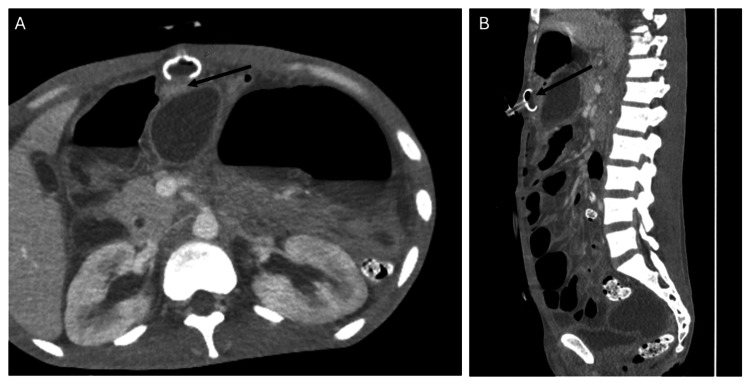
Malposition of a Percutaneous Gastrostomy Tube With Intraperitoneal Placement Non-contrast CT of the Abdomen Demonstrating Malposition of a PEG Tube (A) Axial view shows the tube situated anteriorly, outside the gastric lumen (arrow highlights the gap). (B) Sagittal reconstruction confirms extragastric location of the tube (arrow highlights the gap), with peritoneal enhancement and free fluid suggesting peritonitis from continued tube use. Early recognition of extragastric placement is critical to prevent ongoing leakage, sepsis, and other complications.

A 32-year-old man who suffered from a stroke three weeks prior required placement of a PEG tube. On CT, there was evidence of PEG perforation of the peritoneum due to enhancing peritoneal margins and free peritoneal fluid (Figure 13).

Nasogastric tubes

Representative cases included tracheobronchial misplacement into the right mainstem bronchus and perforation of the gastric wall with subsequent pneumoperitoneum.

**Figure 14:**
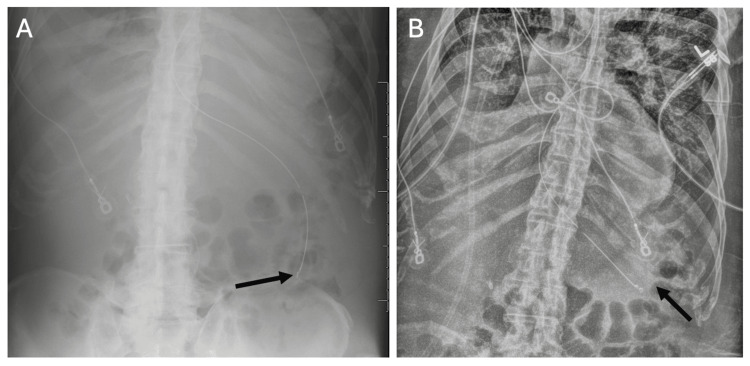
Correct Positioning of a Nasogastric (NG) Tube on Radiographs Radiographs demonstrating appropriate placement of a nasogastric tube. (A) NG tube coursing midline through the esophagus, crossing the diaphragm, and terminating within the gastric body (arrow). (B) Distal tip projecting over the stomach, below the left hemidiaphragm (arrow). Proper recognition of these features is essential to distinguish correct intragastric positioning from esophageal or airway malposition.

Placement involves passing a flexible tube through the nasal passage, past the pharynx, down the esophagus, and into the stomach, with external fixation at the nares to maintain position and prevent migration [13]. On imaging, the NG tube, visible as a radiopaque line, should descend past the carina without bifurcating, indicating it has correctly descended through the esophagus. The tube should also descend past the left hemidiaphragm, terminating in the gastric body or antrum (Figure 14).

If the insertion length is too short, the tip will end up in the esophagus, which can result in aspiration and increase the risk of aspiration pneumonia. Conversely, if the insertion length is too long, the tip can end up in the duodenum, or it can kink in the stomach and curl upwards towards the esophagus [13]. 

Case Example

**Figure 15:**
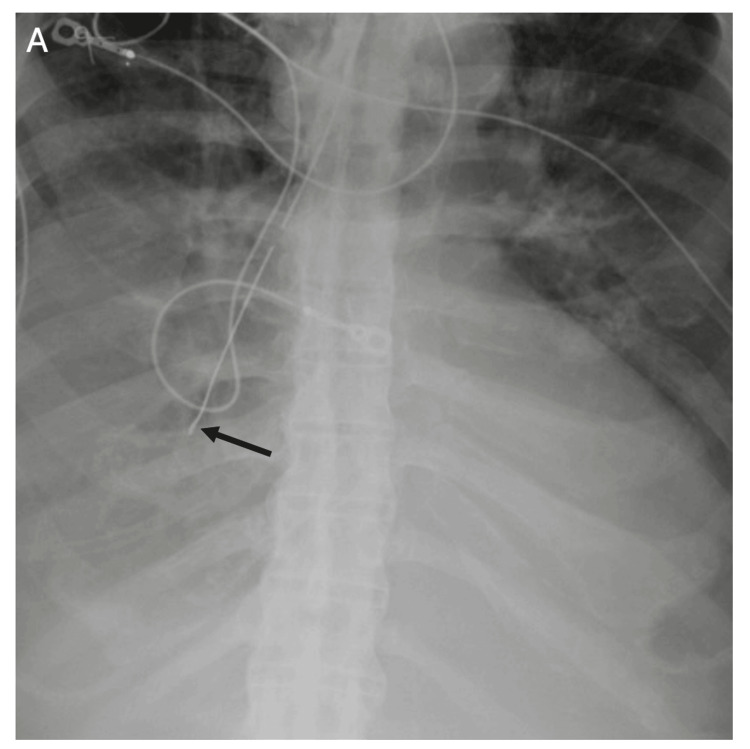
Nasogastric Tube Malposition Into the Right Main Bronchus Radiograph demonstrating malposition of a nasogastric tube into the airway. The tube is seen deviating into the right main bronchus (arrow) rather than crossing the diaphragm into the stomach. Airway malposition is more common on the right due to its more vertical orientation and carries a high risk of aspiration, pneumothorax, or feeding-related complications.

An 89-year-old man who experienced severe respiratory distress required interval removal of an NG tube. Compared to prior imaging, the enteric tube projected over the right mainstem bronchus, and the tip was located over the expected region of the bronchus intermedius (Figure 15), suggesting that the end of the NG tube has been malpositioned into the right bronchus.

Case Example 2

**Figure 16:**
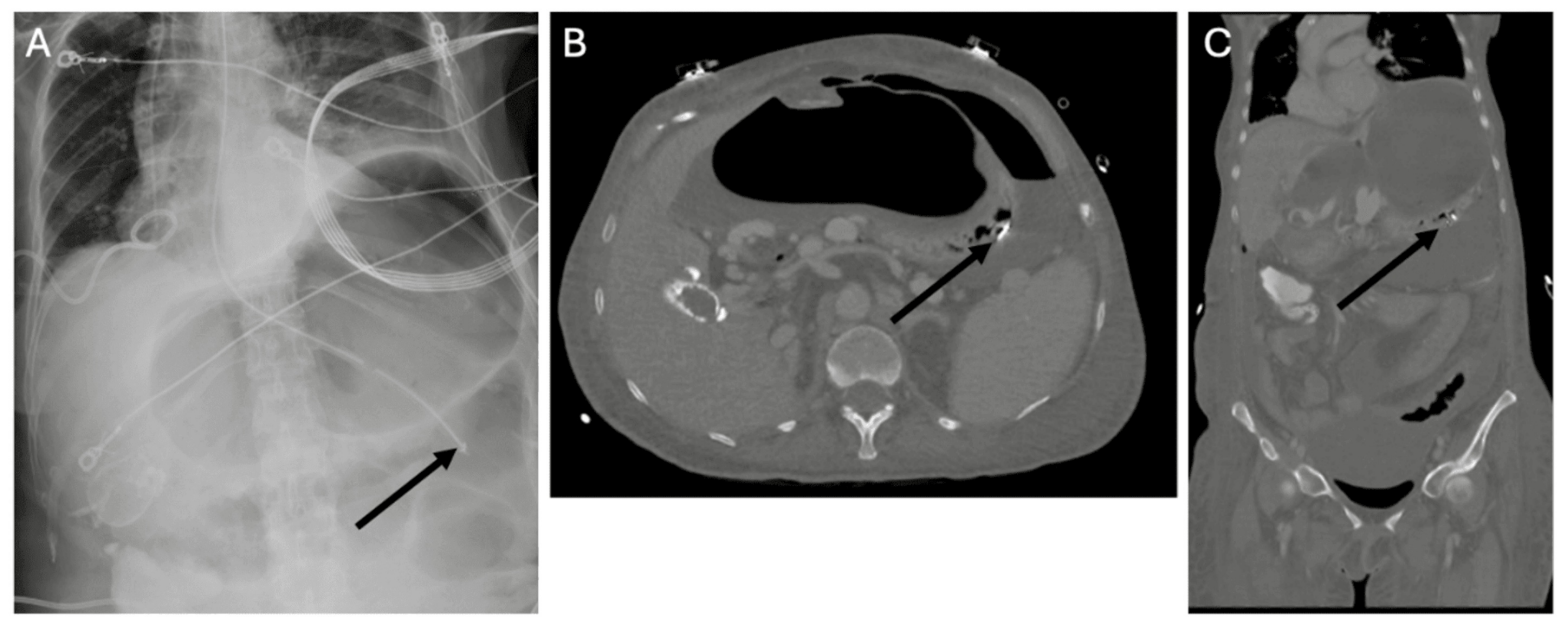
Nasogastric Tube Malposition With Suspected Gastric Wall Erosion and Perforation Imaging of a malpositioned nasogastric (NG) tube with evidence of complication. (A) Chest radiograph shows the NG tube coursing below the diaphragm with the distal tip projecting over the left upper quadrant (arrow) seemingly in the correct space. (B, C) Axial and coronal non-contrast CT images demonstrate the tube tip abutting and extending beyond the gastric wall, with associated free intraperitoneal air and fluid (arrows), concerning for gastric wall erosion and perforation. Prompt recognition is critical, as continued feeding through a malpositioned tube may exacerbate peritonitis and sepsis.

A 64-year-old woman, three weeks post-colorectal fistula takedown, presented with worsening left upper abdominal pain, lower extremity edema, and new-onset ascites. Interval study imaging revealed the patient's NG tube outside the gastric lumen, potentially perforating the stomach mucosa. The consequent enlargement of intra-abdominal air and fluid collection on radiograph, which increased in size from 18.8 × 9.6 cm to 21.5 × 13.5 cm over the course of two days, raised concern for potential malposition, resulting in a pneumoperitoneum. Follow-up radiographs and CT imaging showed the NG tube overlying the upper mediastinum, with layering pleural effusion and a persistent, large gas collection in the upper left quadrant (Figure 16). This radiographic evidence suggested an ongoing intra-abdominal pathology, likely exacerbated by NG tube perforation.

Together, these cases highlight both common and rare presentations of device malposition. Each illustrates the critical role of radiologists in recognizing abnormal positioning and preventing diagnostic delays.

## Discussion

This pictorial case series highlights recurrent mechanisms by which indwelling devices become malpositioned, such as unfavorable anatomic vectors at insertion, technique or verification gaps at placement, and time-dependent migration during dwell. We emphasize a practical, imaging-first strategy that radiologists can apply across devices, followed by device-specific nuances and management priorities: (1) structured verification of expected course and tip location on initial imaging, with early escalation to targeted ultrasound, fluoroscopy, or CT when the trajectory is atypical or the clinical course is discordant; and (2) proactive device stewardship with daily necessity checks, awareness of dwell time, and timely exchange or retrieval when the original indication resolves.

Central venous catheters - upper extremity

For thoracic CVCs, ideal tip placement is in the SVC or at the cavoatrial junction, yet malposition remains common, particularly with left-sided or subclavian approaches [14,15]. Aberrant courses into the azygos, internal mammary, intercostal, or pericardiophrenic veins are recognizable on radiographs by atypical mediastinal or parasternal trajectories [16]. Because arterial cannulation and other serious complications may not be apparent without imaging, procedural cues (aberrant wire behavior, non-physiologic waveforms) should prompt immediate reassessment. Best practice is to stop use, confirm position, and reposition or exchange over a wire rather than rely on blind manipulation; ultrasound wire tracking, fluoroscopy, or ECG-based tip confirmation can further reduce uncertainty at the time of placement [15,16,3].

Central venous catheters - lower extremity

When upper-extremity routes are not feasible, femoral access remains valuable but carries higher infectious and thrombotic risk than internal jugular or subclavian sites [17,18]. Imaging should confirm a straight intraluminal course with the tip in the common iliac vein or IVC. New ipsilateral limb swelling, catheter dysfunction, or refractory alarms should trigger reassessment (often with duplex) for DVT or malposition. Given the differential risk profile, teams should plan for early transition to a thoracic site once the patient's condition allows, alongside standardized line-care bundles to minimize dwell time [17,18].

Ureteral stents

Correct positioning places the proximal coil in the renal pelvis and the distal coil in the bladder; migration presents as a distal coil below the pubic symphysis or a proximal tip beyond the renal contour. Persistent hydronephrosis, flank pain, fever, or recurrent UTIs despite placement should raise suspicion for malfunction. Noncontrast CT or targeted ultrasound clarifies obstruction, periureteral inflammation, encrustation, or an extraluminal course when radiographs are equivocal. Management priorities include prompt urologic evaluation, relief of obstruction, and exchange or repositioning; counseling on dwell time is essential as migration, encrustation, and infection increase with longer indwelling durations [4,19-22].

Despite design improvements aimed at minimizing migration, stent complications remain a common occurrence. Up to 80% of patients experience discomfort, and stents are associated with urinary tract infections (colonization rates: 42-90%) and encrustation (9-76%, depending on duration) [19]. Migration occurs in 8-10% of cases, often due to improper stent length or shape memory of the material. Stents may be placed retrograde via cystoscopy or antegrade through a percutaneous approach, with fluoroscopy typically used to confirm placement [20]. Complication rates for migration (~8%), encrustation (27-76%), and infection (4-26%) all rise with longer stent dwell times (Table 1).

**Table 1:** Complication Rates of Ureteral Stents by Indwelling Duration Reported rates of ureteral stent–related complications stratified by indwelling duration. Longer indwelling times are associated with a sharp rise in encrustation and infection, with migration typically occurring after three months [12,13].

Complication	< 6 weeks	6–12 weeks	> 12 weeks	Key Point
Migration	—	—	~8%	Reported only for longer indwelling; most occur in months 3–12
Encrustation	26.8%	56.90%	75.9%	Sharp increase after 6 weeks, especially beyond 3 months
Infection	4.30%	15–26%	26%	Risk triples after 1 month of indwelling

Ventriculoperitoneal shunts

A structured “shunt series” plus cranial imaging distinguishes proximal under-drainage from distal mechanical issues such as kinking, disconnection, loculated collections, or rare visceral erosions/extrusions. Correlating symptoms (headache, lethargy, emesis, abdominal pain) with imaging supports timely neurosurgical consultation for revision or externalization; broad-spectrum antibiotics are considered when infection or visceral perforation is suspected [23].

Inferior vena cava filters

After deployment, documentation should include device type, level, tilt, patency, and strut-caval wall relationship. Although major complications are uncommon with proper technique, perforation (penetration >3 mm) is not rare and can involve adjacent organs. When the indication resolves, timely retrieval limits long-term device-related issues; cases with marked tilt, embedded apices, or penetrating struts merit referral to centers with advanced endovascular support [24,25].

Percutaneous endoscopic gastrostomy tubes

Complications range from peristomal infection and leakage to malposition with peritonitis. If the clinical course is atypical or position is uncertain, withhold feeds until imaging confirms intragastric placement (internal bumper apposition with intragastric contrast). Early dislodgement before tract maturation carries heightened risk and warrants urgent GI or IR-guided replacement. Fluoroscopy remains reliable for confirmation, with point-of-care ultrasound increasingly used to assess balloon location when radiography is not immediately available [8-12].

Nasogastric tubes

Because airway misplacement can be catastrophic, a properly obtained and interpreted radiograph remains the most dependable method of confirmation [13]. Right-sided airway malposition is more common due to bronchial orientation; intracranial passage and perforations are rare but severe. Any discordance between the tube course and the expected gastric position mandates immediate cessation of use and repeat confirmation [26,27].

Clinical implications

Embedding explicit tip location and course language in radiology reports (“tip at cavoatrial junction; course consistent with right IJ to SVC”) and flagging atypical or indeterminate courses with a recommended next step can shorten time to recognition and intervention. Likewise, early consult pathways in report impressions (e.g., urology for migrated stents; neurosurgery for distal VP shunt extrusion; IR clinic for filter retrieval candidacy) help translate imaging findings into action.

Limitations and future directions

This single-center retrospective series was intentionally didactic and not designed to estimate incidence or compare techniques. Future work should test standardized reporting checklists and evaluate whether predefined escalation triggers reduce delays and complications. Although automation may eventually assist with real-time trajectory checks and tip detection, success will continue to depend on protocol discipline and interdisciplinary handoffs, not automation alone.

In summary, reliable recognition of malposition is less about memorizing every variant and more about applying a structured search pattern - verify the expected path, confirm the tip, identify red-flag trajectories, and escalate early when images and clinical behavior diverge. Coupled with device stewardship, this approach can help prevent harm across these diverse devices.

## Conclusions

Indwelling catheters, stents, and medical devices are increasingly common with the shift toward minimally invasive procedures, and radiologists play a pivotal role in recognizing malposition and migration. These complications, if undetected, may lead to serious outcomes including hemorrhage, infection, obstruction, or perforation. Accurate imaging assessment requires more than confirming device presence; it demands a systematic approach that verifies expected pathways, tip location, and potential abnormal trajectories, supplemented by comparison with prior studies and correlation with clinical findings.

The illustrative cases presented highlight both typical and rare malpositions, underscoring the need for structured search patterns, routine verification of indwelling devices, and judicious use of advanced imaging when radiographs are inconclusive. Looking forward, emerging technologies such as artificial intelligence may enhance real-time recognition, but radiologic expertise and interdisciplinary collaboration remain central to improving diagnostic accuracy, ensuring device safety, and optimizing patient outcomes.
